# Quantitating the effect of prosthesis design on femoral remodeling using high‐resolution region‐free densitometric analysis (DXA‐RFA)

**DOI:** 10.1002/jor.23536

**Published:** 2017-04-13

**Authors:** Mohsen Farzi, Richard M. Morris, Jeannette Penny, Lang Yang, Jose M. Pozo, Søren Overgaard, Alejandro F. Frangi, Jeremy Mark Wilkinson

**Affiliations:** ^1^ University of Sheffield, Academic Unit of Bone Metabolism Northern General Hospital Sheffield United Kingdom; ^2^ Department of Electronic and Electrical Engineering Centre for Computational Imaging & Simulation Technologies in Biomedicine (CISTIB) University of Sheffield Sheffield United Kingdom; ^3^ Department of Orthopaedic Surgery and Traumatology Odense University Hospital University of Southern Denmark Institute of Clinical Research Odense Denmark

**Keywords:** hip arthroplasty, bone mineral density, strain‐adaptive bone remodeling, dual energy X‐ray absorptiometry (DXA), false discovery rate (FDR)

## Abstract

Dual energy X‐ray absorptiometry (DXA) is the reference standard method used to study bone mineral density (BMD) after total hip arthroplasty (THA). However, the subtle, spatially complex changes in bone mass due to strain‐adaptive bone remodeling relevant to different prosthesis designs are not readily resolved using conventional DXA analysis. DXA region free analysis (DXA RFA) is a novel computational image analysis technique that provides a high‐resolution quantitation of periprosthetic BMD. Here, we applied the technique to quantitate the magnitude and areal size of periprosthetic BMD changes using scans acquired during two previous randomized clinical trials (2004 to 2009); one comparing three cemented prosthesis design geometries, and the other comparing a hip resurfacing versus a conventional cementless prosthesis. DXA RFA resolved subtle differences in magnitude and area of bone remodeling between prosthesis designs not previously identified in conventional DXA analyses. A mean bone loss of 10.3%, 12.1%, and 11.1% occurred for the three cemented prostheses within a bone area fraction of 14.8%, 14.4%, and 6.2%, mostly within the lesser trochanter (*p* < 0.001). For the cementless prosthesis, a diffuse pattern of bone loss (−14.3%) was observed at the shaft of femur in a small area fraction of 0.6% versus no significant bone loss for the hip resurfacing prosthesis (*p* < 0.001). BMD increases were observed consistently at the greater trochanter for all prostheses except the hip‐resurfacing prosthesis, where BMD increase was widespread across the metaphysis (*p* < 0.001). DXA RFA provides high‐resolution insights into the effect of prosthesis design on the local strain environment in bone. © 2017 The Authors *Journal of Orthopaedic Research* published by Wiley Periodicals, Inc. on behalf of Orthopaedic Research Society. J Orthop Res 35:2203–2210, 2017.

Prosthesis design influences the local mechanical environment of the proximal femur after total hip arthroplasty (THA), resulting in strain‐adaptive bone remodeling.^1^, ^2^, ^3^ Several factors influence the extent of bone loss that occurs around different prosthesis types; including prosthesis geometry, material stiffness, method of fixation, and surface coating.^4^, ^5^, ^6^, ^7^, ^8^, ^9^, ^10^ Periprosthetic bone loss is a risk factor for fracture and causes reconstruction challenges at revision surgery.^11^, ^12^ Dual energy X‐ray absorptiometry (DXA) is the reference standard method used to study bone mineral density (BMD) after THA.^13^, ^14^However, the resolution of conventional DXA analysis is a limiting factor as spatial information is lost by pooling pixels into a small number of pre‐defined regions of interest (ROIs)^15^, ^16^ that substantially limit the precise localization and quantitation of BMD change events. This data averaging also leads to inconsistent results for a given dataset depending on the number and placement of the analysis ROIs.^5^, ^15^, ^17^, ^18^, ^19^, ^20^

There is a need for high‐resolution, low‐radiation exposure technologies for evaluating the bone architectural changes associated with different biomaterial designs and implant geometries.^21^ Such technologies would facilitate the non‐invasive clinical assessment of novel prostheses that aim to better mimic the natural loading environment, or have surface coatings that aim to modulate the biology of the local bone environment.^22^ We recently reported a high‐resolution computational method for DXA scan analysis, termed DXA region free analysis (RFA).^23^ DXA RFA applies current advances in image processing, non‐rigid registration, and statistical parametric mapping to quantitate BMD at the individual pixel‐level.^24^, ^25^, ^26^ The DXA RFA method enables quantitation of the areal size and the anatomic position of regions with statistically significant BMD change without imposing any a‐priori assumptions on the analysis region of interest. To this end, we have extended the DXA RFA tool to control for statistical error rates in multiple tests using the False Discovery Rate method (FDR) to enable comparative inferences to be drawn.^27^ This approach has previously been applied to femoral cortical bone analysis using quantitative computed tomography images,^28^ in functional neuroimaging,^29^ and in similarly large datasets in other fields.^30^, ^31^

Here, we applied the extended DXA RFA method to examine the impact of prosthesis design on strain‐adaptive bone remodeling in the setting of two previously reported clinical trials using substantially different femoral prosthesis designs.^32^, ^33^ In one trial, we compared three different geometries of cemented femoral prosthesis, the Charnley (DePuy International, Leeds, UK), Exeter (Stryker, Newbury, UK), and the C‐Stem (DePuy International, Leeds, UK). These prostheses may be classified as shape‐closed or force‐closed designs.^34^, ^35^ Shape‐closed designs, like the Charnley, use a bonded prosthesis‐cement interface to fix the stem within the cement mantle, acting as a composite‐beam, and transfer load to the femur mainly at the level of femoral diaphysis. Force‐closed designs, such as the double‐tapered (Exeter) and triple‐tapered (C‐Stem) prostheses, have a non‐bonded prosthesis‐cement interface, where the stem acts as a mobile wedge within the cement mantle.^34^, ^36^ This allows initial distal migration to set up hoop stresses in the proximal cement mantle resulting in more proximal load transfer between the femoral prosthesis and the host bone.^37^ In the other trial, we compared bone remodeling around a hip resurfacing prosthesis versus a conventional cementless total hip replacement. The load transfer pattern in RHR occurs directly from the femoral head to the metaphysis, and is thought to be more representative of that found in the native proximal femur than that for a conventional stemmed prosthesis.^20^, ^38^, ^39^, ^40^, ^41^

## MATERIALS AND METHODS

### Study Populations and Scan Acquisitions

Anonymized DXA scans from two previous ethically approved clinical trials, for which written, informed consent was provided, were examined using DXA RFA.^32^, ^33^ All subjects underwent surgery for idiopathic or secondary osteoarthritis, and were free from use of drugs known to affect BMD. All scans were acquired using a Hologic QDR 4500A fan‐beam densitometer (Hologic Inc., Bedford, MA), using the “metal removal hip” scanning mode with a point resolution of 0.6 mm and a line spacing of 1.1 mm. Scans were performed with the subject in the supine position with the legs in neutral rotation and full extension. Scan acquisition was started approximately 2.5 cm distal to the tip of the femoral prosthesis, with the longitudinal axis of the prosthesis shaft vertical and occupying the center of the scan field. The scan was continued proximally until 2 cm above the tip of the greater trochanter.^15^

### Study Designs and Subject Monitoring

#### FDR Validation

To investigate the accuracy of FDR algorithm incorporation into the DXA RFA framework, we examined sequential DXA scans taken on the same day after repositioning in 17 men (mean age 50 years, range 33–67) and 12 women (mean age 53 years, range 35–61). Scans were acquired a mean of 6 months (SD 3) after THA.^15^ The hypothesis tested here was that we expected no significant differences in measured pixel‐level BMD between the individual scan pairs at FDR level of 0.05.

#### The Effect of Cemented Stem Design on Bone Remodeling

The subjects in this study were randomized at a ratio of 1:1:1 to receive either a cemented composite‐beam prosthesis (Charnley, DePuy Synthes Ltd, *n* = 35), a double‐tapered prosthesis (Exeter, Stryker UK Ltd, *n* = 38), or a triple‐tapered prosthesis (C‐stem, DePuy Synthes Ltd, *n* = 38).^32^ All patients were mobilized with unrestricted weight bearing on the first or second post‐operative days. BMD was measured at post‐operative baseline within 1 week of surgery, and at 3, 6, 12, and 24 months later using the same Hologic densitometer.

#### Effect of Hip Resurfacing Versus Cementless THA on Bone Remodeling

The subjects in this study were randomized at a ratio of 1:1 to receive either a hip resurfacing prosthesis (Articular Surface Replacement (ASR) total femoral prosthesis, DePuy Synthes Ltd, *n* = 13) or THA using a cementless, proximally plasma‐coated, titanium femoral component (Bi‐metric, Biomet, Bridgend, UK, *n* = 17).^33^ All patients were mobilized full weight bearing on the first or second post‐operative days. BMD was measured at post‐operative baseline within 1 week of surgery, and at 2, 12, and 24 months later using the same Hologic densitometer.

### Scan Analysis

The DXA‐RFA method was based upon a proprietary DXA bone map extraction algorithm APEX 3.2 (Hologic Inc, Waltham, MA), and implemented in Matlab v7.11.0.584 r2010b (Mathworks Inc, Cambridge, MA), and performed as previously described.^23^

#### Image Segmentation

Briefly, for each Hologic prosthetic hip scan BMD image of the proximal femur was extracted from the two archived Hologic scan files using DXA‐RFA (.p and .r files, approximately 14,000 pixels per scan; mean pixel size 0.56 × 0.56mm^2^). The extracted images were then segmented into prosthesis, bone, and soft tissue compartments using edge‐detection, intensity thresholding, and morphological operations. Subsequently, the pixel BMD values within the bone compartment were computed using DXA‐RFA.

#### Image Alignment and Template Registration

Anatomic landmark and control points were defined automatically for each DXA scan, as previously described.^23^ Next, separate scan templates were generated for each prosthesis type using the Generalized Procrustes algorithm.^42^ For each prosthesis type, the individual scans were registered to the corresponding template using a thin plate spline (TPS) algorithm.^26^

#### Baseline Analysis

The baseline demographic characteristics of the subjects between each of the prosthesis groups were compared using the χ^2^ test, Fisher's exact test, the Mann–Whitney *U* test, or Student's *t*‐test, as appropriate. The mean distribution of pixel BMD values among the post‐operative baseline scans was computed for each prosthesis.

#### False Discovery Rate Analysis

The pixel‐level BMD changes with respect to the baseline measurement were examined using a paired *t*‐test at each time‐point. Next, to address the multiple testing issue, the FDR was controlled using the Benjamini and Hochberg approach.^43^ In this approach, the acceptable rate α is defined beforehand (here at 0.05) and the corresponding *p*‐value threshold is then estimated. This method selects the set of pixels with significant BMD change at FDR level α, yet does not provide corrected *p*‐values for each pixel. The FDR analogue to the *p*‐value is called *q*‐value. The *q*‐value of a pixel is the minimum FDR level α for which this pixel is selected as significant. The mapping from *p*‐values to *q*‐values is obtained as follows. First, the *p*‐values are sorted increasingly as $p_{\left(1\right)},\leq p_{\left(2\right)}\leq \ldots \leq p_{\left(N\right)}$. The corresponding *q*‐values are then given by $q_{\left(i\right)}=p_{\left(i\right)}*\frac{N}{i}$.

All pixels with q≤0.05 were selected as statistically significant. The areal size of regions with significant BMD change was quantitated as the fraction of periprosthetic bone area, that is, the number of pixels with q≤0.05 divided by the number of all pixels in the template. The areal proportions were then compared between prosthesis designs using a chi‐squared test. The pixel‐level FDR *q*‐values were also rendered as heat‐maps to denote the anatomic location of significant BMD change events within the bone.

## RESULTS

### FDR Validation

**Figure 1:**
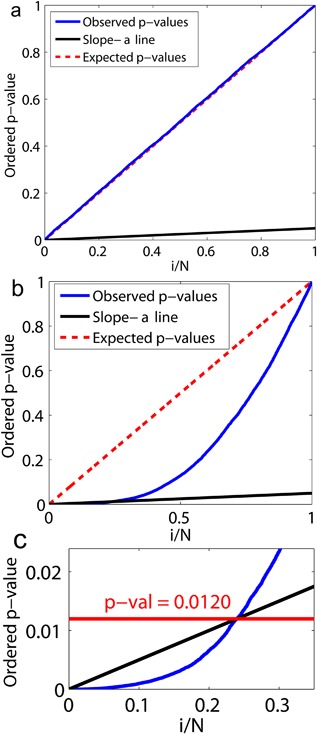
P‐P plot for FDR analysis. (a) The P‐P plot for the set of 29 repositioned pairs of scans. As shown, the blue line almost perfectly follows the diagonal line of identity indicating that the null hypothesis of no change is valid in all pixels. (b) The P‐P plot for Charnley prosthesis after 24 months. The blue line deviates below the line of identity, indicating the rejection of null hypothesis. (c) All pixels below the slope‐ α line corresponding with *p*‐value less than 0.012 are statistically significant at α=0.05.

Figure 1 shows the P‐P plots for the repositioned scans examined here. A P‐P plot is a diagram of increasingly sorted observed *p*‐values against the i/(N+1) quantile of the uniform distribution, where N is the total number of observed *p*‐values. Under null hypothesis, the expected curve in the P‐P plot is the diagonal line of identity. Large deviations from this diagonal have lower probability. As shown in Fig. 1a, the P‐P plot follows the line of identity. This means that no pixels with significant BMD change were identified across all pixels in the 29 subject pairs, confirming the null hypothesis. In comparison, Fig. 1b shows the P‐P plot for the Charnley prosthesis after 24 months as an example where the null hypothesis is rejected, since the P‐P plot deviates below the slope‐ α line (Fig. 1c).

### Clinical Trial Subject Characteristics

The participants within each clinical trial were of similar age, sex distribution, and body mass index (Table 1). The subjects participating in the cemented stem geometry trial were older than those participating in the conventional cementless femoral prosthesis versus hip resurfacing trial (71 ± 6 vs. 57 ± 6, *p *< 0.001), and a greater proportion were female (53:58 vs. 22:8, *p* = 0.013). The BMI of participants in each study were 29.2 ± 4.4 versus 28.3 ± 4.4, respectively (*p* = 0.397).

**Table 1:** Characteristics of the Patient Populations Participating in the DXA RFA Analyses

Cemented Femoral Stem Geometry Study					
Characteristic	Charnley (*n* = 35)	C‐Stem (*n* = 38)		Exeter (*n* = 38)	*p*‐Value
Age at surgery (years)	70 ± 6	71 ± 7		71 ± 6	^a^0.929
Sex (M:F)	14:21	19:19		20:18	^c^0.527
BMI (kg/m^2^)	28.9 ± 4.6	29.2 ± 4.8		29.3 ± 3.9	^a^0.914
–					
Cementless Stemmed Versus Hip Resurfacing Study					
Characteristic	Hip Resurfacing (*n *= 13)		Cementless Stem (*n *= 17)		*p‐V*alue
Age at surgery (years)	57 ± 6		56 ± 6		^b^0.320
Sex (M:F)	8:5		14:3		^d^0.201
BMI (kg/m^2^)	28.0 ± 5.9		28.6 ± 3.0		^b^0.680

Continuous data are presented as mean ± standard deviation, and analysis is between groups within each study using ^a^ANOVA or ^c^Mann–Whitney test. Categorical data were analyzed using the ^b^chi‐squared or ^d^Fisher's exact test.

### Post‐Operative Baseline Mean BMD Distribution

**Figure 2:**
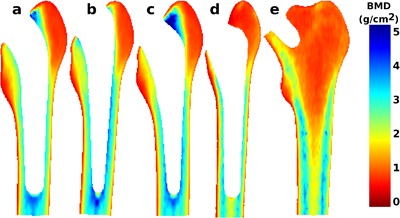
Mean pixel BMD distribution. The mean distribution of pixel BMD values at baseline measurement is shown for (a) composite‐beam (Charnley), (b) double‐taper (Exeter), (c) triple‐taper (C‐stem), (d) Bi‐Metric total hip replacement, and (e) ASR hip resurfacing prosthesis designs, respectively.

Baseline scans for all prosthesis groups showed a pattern of mean BMD distribution consistent with proximal femoral architecture with differentiation of cancellous versus cortical bone (Fig. 2). Areas of lowest BMD (approximately, 0.5–1g/cm^2^) were observed in the cancellous bone within the greater and lesser trochanter. BMD was highest (2–3g/cm^2^) in the cortical bone of the femoral diaphysis. Subjects with cemented prostheses showed highest bone mass in the region of cementation, with a measured BMD of up to 4g/cm^2^.

### Effect of Cemented Stem Design on Bone Remodeling

**Figure 3:**
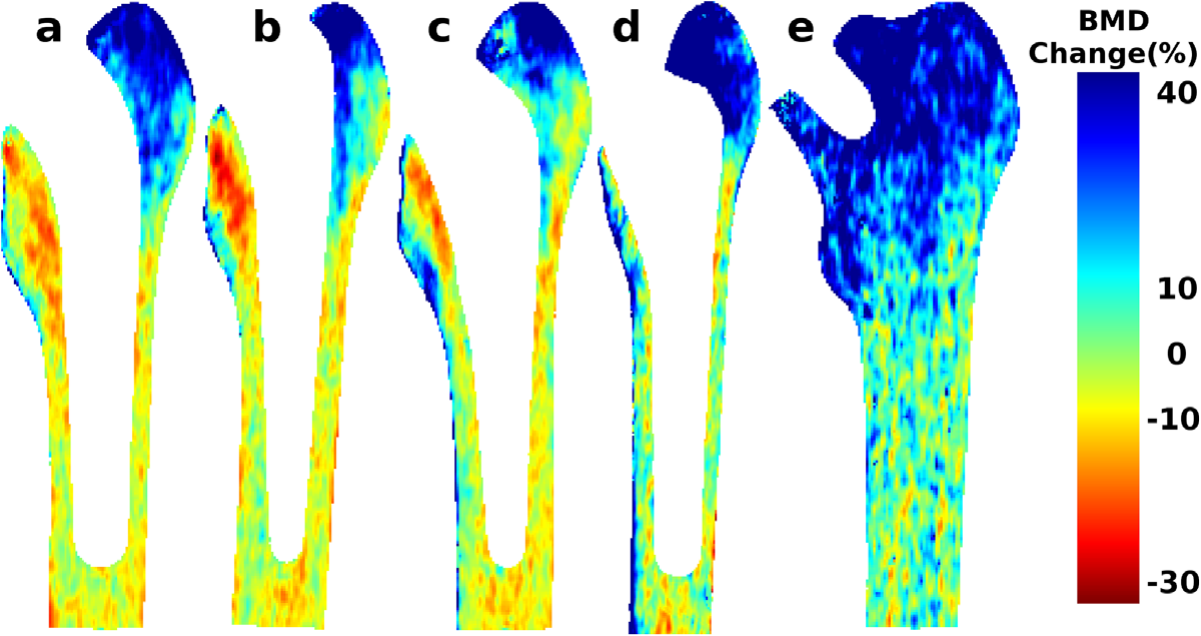
Longitudinal mean pixel BMD change over 24 months. The pixel BMD change after 24 months is expressed as a percentage of the baseline measurement. The mean distribution of pixel BMD change after 24 months is shown for (a) composite‐beam (Charnley), (b) double‐taper (Exeter), (c) triple‐taper (C‐stem), (d) Bi‐metric total hip replacement, and (e) ASR hip resurfacing prosthesis designs, respectively.

**Figure 4:**
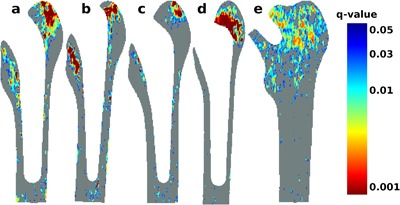
FDR *q*‐value maps after 24 months. The significance of pixel BMD changes is quantitated using the FDR analysis at each pixel. The corresponding *q*‐values are shown for (a) composite‐beam (Charnley), (b) double‐taper (Exeter), (c) triple‐taper (C‐stem), (d) Bi‐Metric total hip replacement, and (e) ASR hip resurfacing prosthesis designs, respectively. All pixels with *q* ≤ 0.05 are declared as significant bone remodeling events in this study.

Some common remodeling features were observed across all the cemented prosthesis designs over the 24‐month trial periods. Figure 3a–c show the magnitude of pixel BMD change (%) at 24 months, and Fig. 4a–c show the corresponding FDR q maps. The percentage bone areas over which a significant change in BMD was observed for each prosthesis by 24 months are shown in Table 2.

**Table 2:** Area Size of Regions With Significant Pixel BMD Change (*q* ≤ 0.05) With Corresponding Mean BMD Change for Three Cemented Prosthesis Designs Over 24 Months

	Total		Increased BMD		Decreased BMD	
	Area Size (%)	Average BMD (%)	Area Size (%)	Average BMD (%)	Area Size (%)	Average BMD (%)
Charnley	31.4	12.2	16.6^a^	32.1	14.8	−10.3
Exeter	24.1	5.3	9.7^a^	31.2	14.4	−12.1
C‐stem	12.7	12.1	6.5^a^	34.5	6.2	−11.1

The area sizes are expressed as a percentage of the total area of periprosthetic bone in the template image. The average BMD change values are also expressed as a percentage of the baseline BMD value. Area of increased BMD comparison between prosthesis designs by chi‐squared test with post‐hoc correction. ^a^ *p *< 0.001.

BMD change events occurred in discrete focal areas. An increase in bone mass was observed consistently in the greater trochanter area, a site of multiple tendinous attachments. Here, an average BMD increase of 32.1% within 16.6% of the periprosthetic bone area was observed for the cemented composite beam (Charnley) prosthesis, 31.2% within 9.7% of the area for the cemented sliding double‐taper (Exeter) prosthesis, and 34.5% within 6.5% of the area for the cemented sliding triple‐taper (C‐stem) prosthesis was observed at 24 months (*q* ≤ 0.05 all comparisons). The areal proportions associated with bone gain were significantly different between the three cemented designs (*p* < 0.001) Table 3.

**Table 3:** Area Size of Regions With Significant Pixel BMD Change (*q *≤* *0.05) With Corresponding Mean BMD Change for a Conventional Cementless Femoral Prosthesis (Bi‐Metric) Versus a Hip Resurfacing Femoral Prosthesis (ASR) Over 24 Months

	Total		Increased BMD		Decreased BMD	
	Area Size (%)	Average BMD (%)	Area Size (%)	Average BMD (%)	Area Size (%)	Average BMD (%)
Cementless stem	22.9	34.6	22.3	35.9	0.6	−14.3
Hip resurfacing	30.7	34.3	30.7	34.3	0.0	0.0

The area sizes are expressed as a percentage of the total area of periprosthetic bone in the template image. The average BMD change values are also expressed as a percentage of the baseline BMD value.

An average bone loss of 10.3%, 12.1%, and 11.1% within an area of size 14.8%, 14.4%, and 6.2% was observed for the Charnley, Exeter, and C‐stem prostheses, respectively (*q* ≤ 0.05), mostly at the lesser trochanter. The areal proportions associated with bone loss were also significantly different between the three cemented designs (*p* < 0.001). The greatest BMD changes occurred in the metaphyseal region for all cemented prosthesis designs, with relatively less change at the femoral diaphysis.

Bone remodeling patterns were both rate and location specific to each prosthesis design (supplementary Figs S1–S3). No significant BMD change was observed at any pixel at 3 months for the Charnley prosthesis. However, an average BMD increase of 12.7% was observed within a small fraction (0.7%) of the periprosthetic bone area for the C‐stem prosthesis at this time‐point (*q* ≤ 0.05), and bone loss of 6.8% over 7% of the bone area medial to the Exeter prosthesis (*q* ≤ 0.05). When we stratified the dataset by subject sex to determine whether this was a significant covariate, we observed a trend toward smaller areas of bone loss and lower magnitude of bone loss in men versus women across the cemented prosthesis designs (Supplementary Table S1).

### Effect of Hip Resurfacing Versus Cementless THA on Bone Remodeling

An average BMD increase of 35.9% over an area of 22.3% was observed locally at the greater trochanter for the Bi‐metric prosthesis (Figs. 3d and 4d; *q* ≤ 0.05). A diffuse pattern of bone loss (−14.3%) was also observed at the shaft of femur for Bi‐metric prosthesis at 24 months over a small fraction of periprosthetic bone area (0.6%). No periprosthetic bone loss was observed around the hip resurfacing prosthesis at 24‐months (Figs. 3e and 4e). However, an average BMD increase of 34.3% was observed over 30.7% of the proximal femoral metaphysis (*q *≤ 0.005). The areal proportions associated with bone gain were significantly different between the hip resurfacing prosthesis and the cementless hip replacement technique (*p* < 0.001).

The contrasting patterns of focal trochanteric versus widespread metaphyseal increase in BMD for the Bi‐metric versus ASR prostheses was apparent by 12 months, and persisted at 24 months (Supplementary Figs. S4 and S5). The increase in bone mass around the ASR prosthesis was observed over the whole proximal femoral metaphysis, but was most densely concentrated in the bone adjacent to the lateral border of the prosthesis and greater trochanter. For the comparison between hip resurfacing versus the cementless THA, the number of women in both prosthesis groups was too small to allow a meaningful stratified analysis by sex.

## DISCUSSION

We analyzed BMD changes around five different prosthesis designs using DXA RFA with FDR to demonstrate in high‐resolution the effect that different prosthesis designs have on proximal femoral strain‐adaptive remodeling. This approach is widely clinically applicable, non‐invasive, and associated with low‐radiation exposure. We observed some remodeling features that were common around all prosthesis, and others that were design‐specific. Our finding that remodeling events occurred in small but spatially discrete “quanta” is consistent with the concept that post‐operative bone remodeling occurs in discrete multicellular units.^44^, ^45^ The observation that periprosthetic bone remodeling events are spatially complex, heterogeneous, and vary in density distribution with prosthesis design supports finite element analysis predictions.^46^ It is also consistent with the view that the conventional ROI‐based approach results in substantial data loss that impacts interpretation.^15^

Consistently across all prosthesis designs, we found a gain in bone mass in the region of the greater trochanter, albeit this increase in bone mass was most widely distributed for the hip resurfacing group. Hip resurfacing was also the only prosthesis design around which increased bone mass occurred within the cortical bone of the proximal medial femur. This aligns with finite element predictions of the stress‐redistribution at the femoral neck induced by this prosthesis class.^47^, ^48^ We have previously identified a similar BMD trend using conventional DXA,^33^ however, analysis using DXA RFA enabled precise localization of the magnitude and area of these events. Although, these data support the concept that head resurfacing prosthesis induce load transfer at the metaphyseal level, the approach does not quantitate over the studied timeframe the possible influence of adverse responses to metal debris on the local tissue microenvironment.

Previous conventional analysis using the seven Gruen zones showed that the greatest bone loss occurred in R7 and R6 over 2 years for the three cemented designs.^32^ While DXA RFA analysis also showed significant bone loss adjacent to the prosthesis at lesser trochanter (Fig. 4a–c), this was more precisely resolved using the technique. Small areas of bone gain at the tendon‐bone interface of the lesser trochanter were also observed (Fig. 3a–c). In conventional DXA analysis, this spatial information is lost due to the averaging pixels into regions of interest. Moreover, this averaging may cancel out the bone loss with the bone gain in a region. The data for the cemented prostheses stratified by subject sex also suggested a smaller magnitude of bone loss and over a small periprosthetic area in men versus women. However, the subject numbers for this comparison were small and should be interpreted with caution. For the hip resurfacing prosthesis, the conventional analysis showed a bone gain in all the Gruen zones.^33^ This is compatible with spatial BMD change patterns in Figure 3e, where these changes are anatomically observed in the femoral shaft. The number of women in the cementless THA versus hip resurfacing study was <10, and a gender‐specific comparison was not performed.

The incorporation of FDR into the DXA RFA framework enabled quantitation of the architectural details of femoral bone mass distribution and robust statistical analysis of BMD change events. These changes were also rendered as heat‐maps for visual assessment. The FDR algorithm was applied to limit the proportion of false positives among statistically significant results. This primary concern is not directly addressed with Bonferroni‐type adjustments.^31^, ^43^ Moreover, the FDR approach gives increased statistical power in comparison with the methods that control family wise error rate.^31^, ^43^ The validation of the FDR correction on the set of 29 repositioned scans confirmed the reliability of the method when applied in the DXA RFA framework.

The DXA RFA analysis approach is also subject to limitations. The method provides a two dimensional representation of three‐dimensional events. However, this is a limitation of DXA per‐se rather than this analysis solution, the principle of which may be applied equally to cross‐sectional imaging as to planar images. DXA RFA uses a template to create an average representation of the femoral anatomy within the study population. We have previously shown that this approach does not affect substantially the precision or accuracy of the tool for femoral bone analyses.^23^

In conclusions, the DXA‐RFA analysis approach shows that bone remodeling after prosthesis insertion occurs in discrete focal quanta that are spatially complex and prosthesis‐specific. This approach provides a low‐radiation exposure method for the radiographic assessment of novel prosthesis designs in the clinical setting, and an opportunity to better enable comparisons between densitometry data, in vivo and in silico biomechanical tools, and other analytical methodologies.

## AUTHOR'S CONTRIBUTIONS

MF, AFF, JMW contributed to research design, JP, SO, JMW contributed to data acquisition, and MF, RMM, LY, JMP, AFF, JMW contributed to data analysis. All authors have drafted for this paper and critical revisions.

## Supporting information

Additional supporting information may be found in the online version of this article.

Supporting Figure S1.

Supporting Figure S2.

Supporting Figure S3.

Supporting Figure S4.

Supporting Figure S5.

Supporting Table S1.

## Appendix

Five images showing the BMD change patterns at intermediate time‐points associated with each prosthesis are available with the online version of this article as a data supplement.
