# Supplementary figures and images for: Quantitating the effect of prosthesis design on femoral remodeling using high‐resolution region‐free densitometric analysis (DXA‐RFA)
Source: J Orthop Res. 2017 Apr 13;35(10):2203–10. doi: 10.1002/jor.23536 (PMC5655934; doi:10.1002/jor.23536)

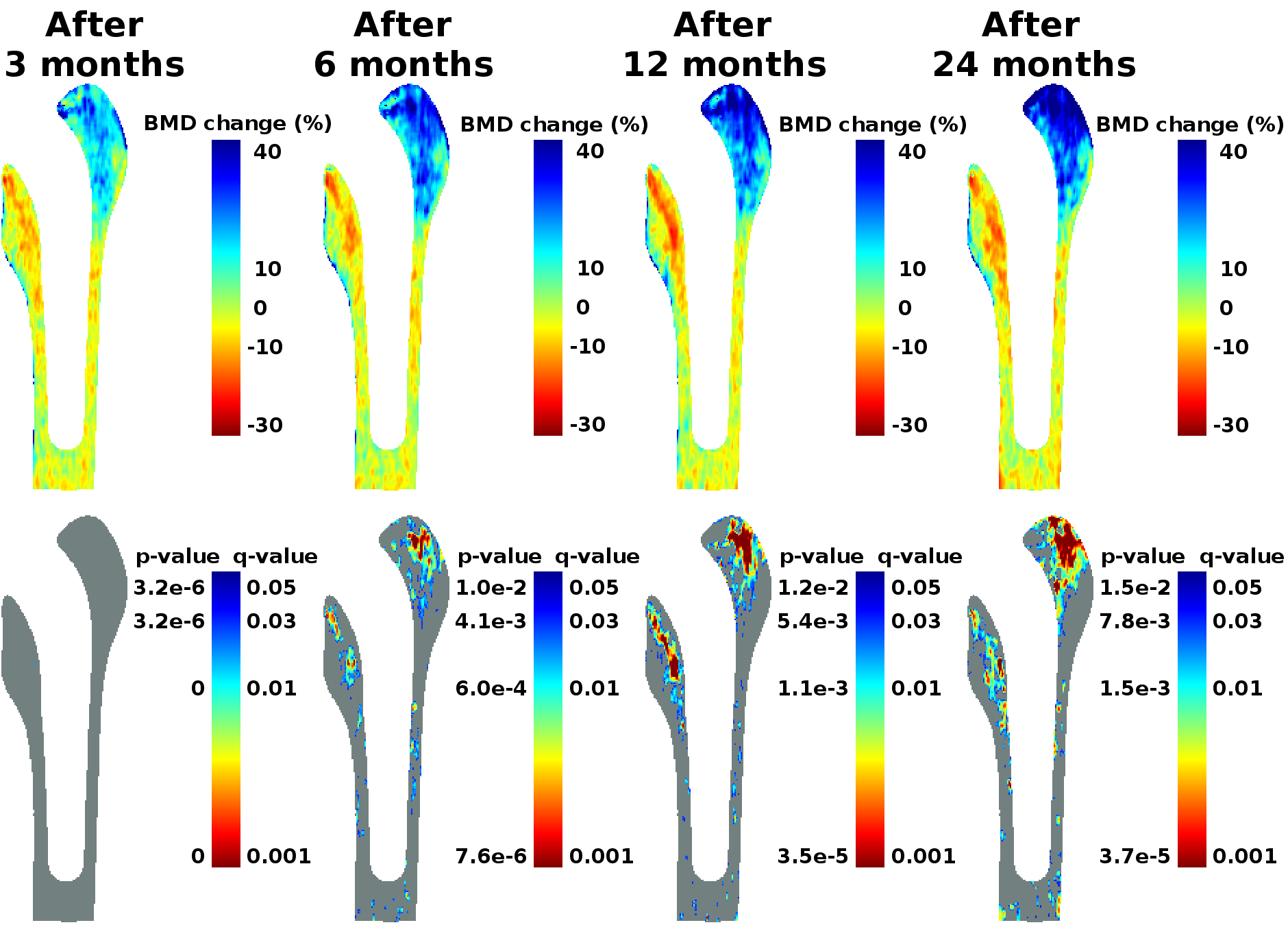

Supplement: Supplementary file 1 — Supporting Figure S1. [file JOR-35-2203-s001.tif]

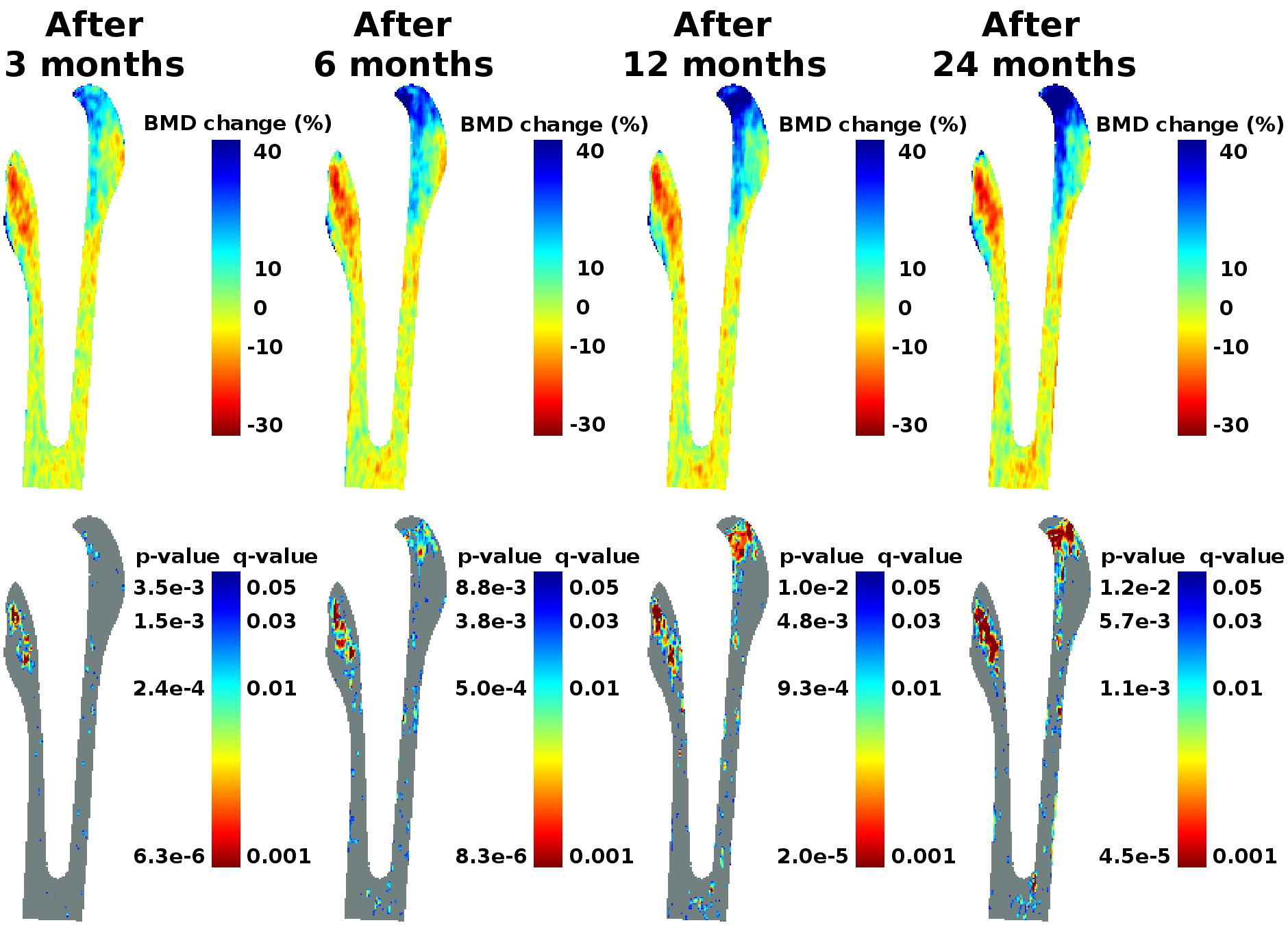

Supplement: Supplementary file 2 — Supporting Figure S2. [file JOR-35-2203-s002.tif]

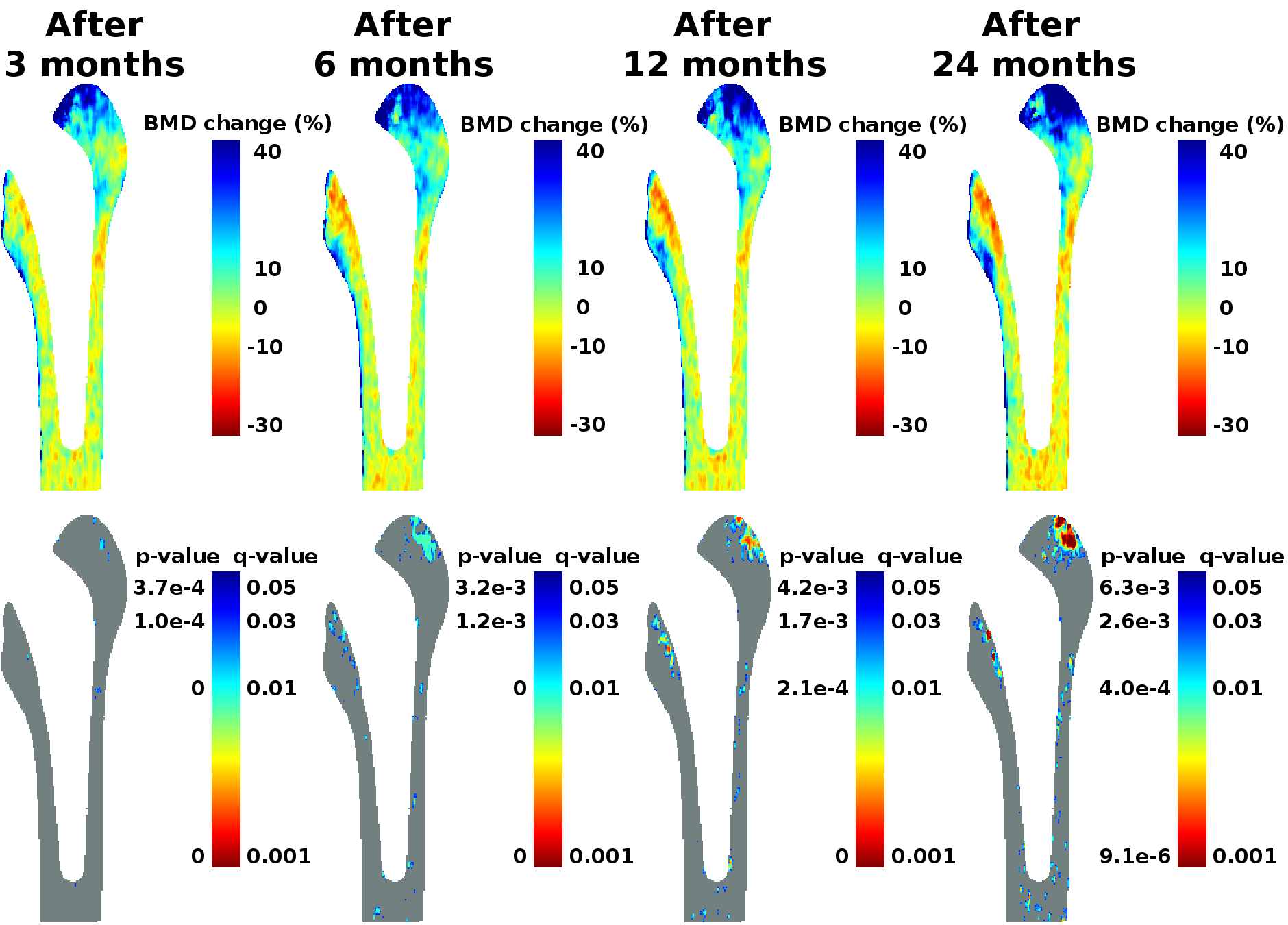

Supplement: Supplementary file 3 — Supporting Figure S3. [file JOR-35-2203-s003.tif]

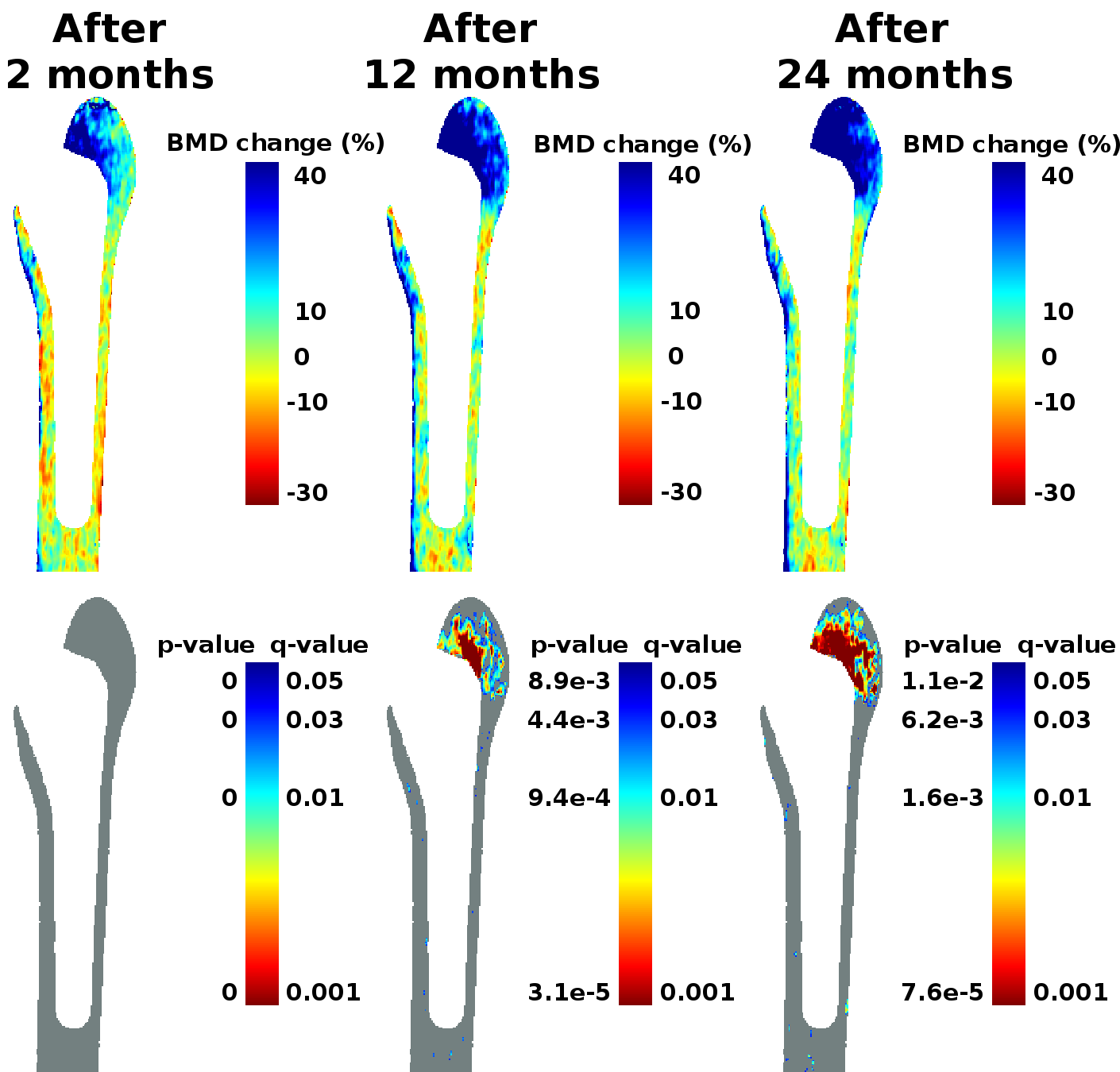

Supplement: Supplementary file 4 — Supporting Figure S4. [file JOR-35-2203-s004.tif]

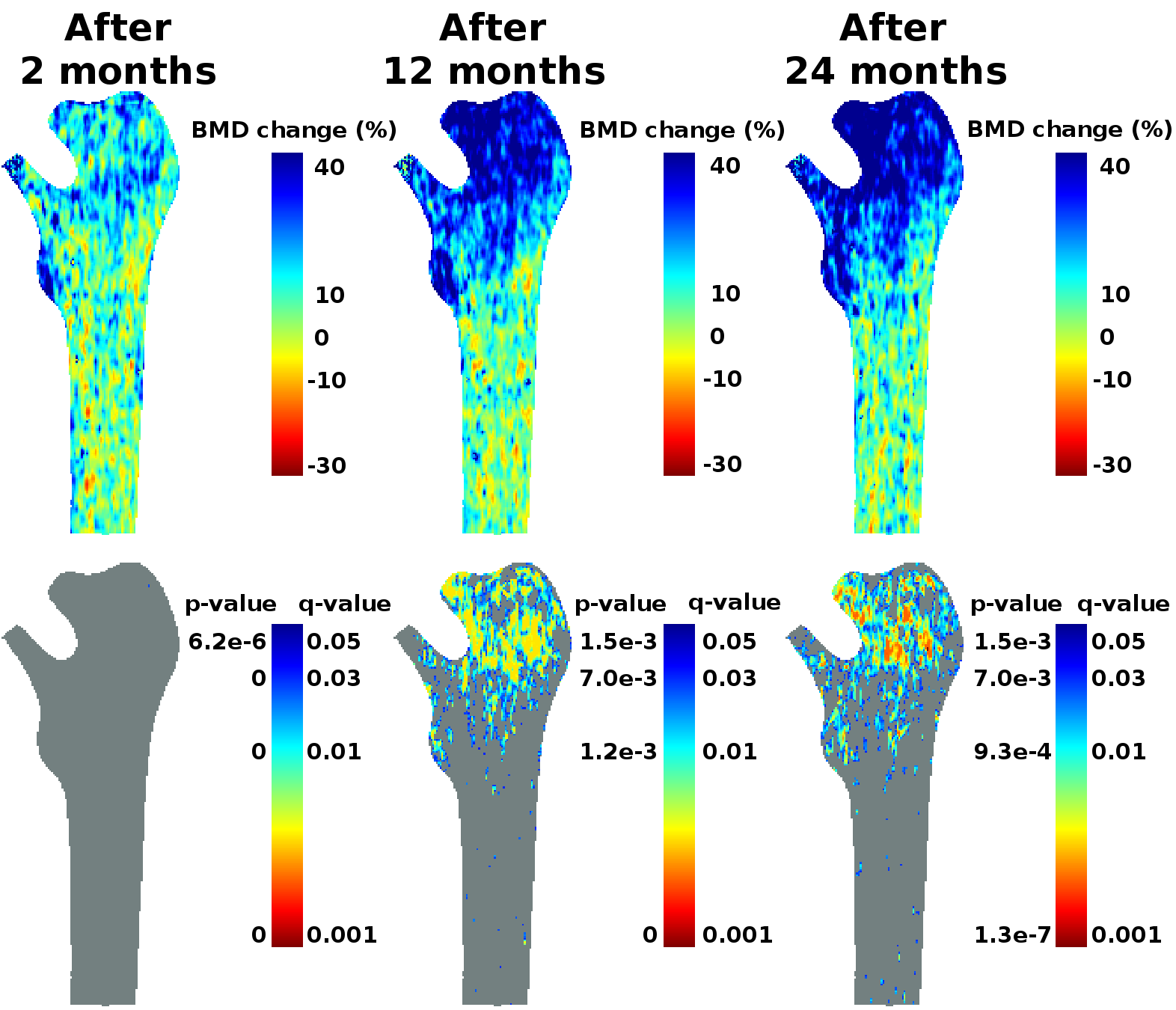

Supplement: Supplementary file 5 — Supporting Figure S5. [file JOR-35-2203-s005.tif]
