# Supplementary material for: Quantitating the effect of prosthesis design on femoral remodeling using high‐resolution region‐free densitometric analysis (DXA‐RFA)
Source: J Orthop Res. 2017 Apr 13;35(10):2203–10. doi: 10.1002/jor.23536 (PMC5655934; doi:10.1002/jor.23536)
Supplement: Supplementary file 6 — Supporting Table S1. [file JOR-35-2203-s006.docx]

|  | | **Total** | | **Increased BMD** | | **Decreased BMD** | |
| --- | --- | --- | --- | --- | --- | --- | --- |
|  |  | Area size (%) | Average BMD(%) | Area size (%) | Average BMD(%) | Area size (%) | Average BMD(%) |
| Charnley | Male (n=14) | 0.0 | 0.0 | 0.0 | 0.0 | 0.0 | 0.0 |
|  | Female (n=20) | 21.0 | 13.4 | 11.7 | 35.1 | 9.3 | -14.2 |
| Exeter | Male (n=20) | 4.8 | 11.0 | 2.6 | 36.7 | 2.2 | -19.4 |
|  | Female (n=17) | 8.3 | -3.1 | 2.4 | 36.0 | 5.9 | -19.1 |
| C-stem | Male (n=17) | 0.3 | 32.0 | 0.3 | 32.0 | 0.0 | 0.0 |
|  | Female (n=17) | 4.0 | -8.1 | 0.9 | 39.8 | 3.1 | -22.0 |

**Supplementary table 1: Area size of regions with significant pixel BMD change (q≤0.05) with corresponding mean BMD change for 3 cemented prosthesis designs over 24 months stratified by subject sex.** The area sizes are expressed as a percentage of the total area of periprosthetic bone in the template image. The average BMD change values are also expressed as a percentage of the baseline BMD value.
